# Implications of Sublethal Insecticide Exposure and the Development of Resistance on Mosquito Physiology, Behavior, and Pathogen Transmission

**DOI:** 10.3390/insects12100917

**Published:** 2021-10-08

**Authors:** Felipe Andreazza, Eugênio E. Oliveira, Gustavo Ferreira Martins

**Affiliations:** 1 Departamento de Entomologia, Universidade Federal de Viçosa, Viçosa 36570-900, MG, Brazil; felipe.andreazza@ufv.br (F.A.); eugenio@ufv.br (E.E.O.); 2 Departamento de Biologia Geral, Universidade Federal de Viçosa, Viçosa 36570-900, MG, Brazil

**Keywords:** host-seeking behavior, insecticide exposure, insecticide resistance, mosquito, pathogen transmission

## Abstract

**Simple Summary:**

Mosquitoes are one of the greatest threats to human lives; they transmit a wide range of pathogens, including viruses that cause lethal diseases. Mosquitoes are found in both aquatic (as larvae or pupae) and terrestrial (as adults) environments during their complex life cycle. For decades, insecticides have been systematically used on mosquitoes with the aim to reduce their population. Little is known about how the stress resulting from the exposure of mosquitoes to insecticides impacts the tri-partite relationship between the mosquitoes, their vertebrate hosts, and the pathogens they transmit. In this work, we review existing experimental evidence to obtain a broad picture on the potential effects of the (sub)lethal exposure of hematophagous mosquitoes to different insecticides. We have focused on studies that have advanced our understanding of their physiological and behavioral responses (including the mechanisms behind insecticide resistance) and the spread of pathogens by these vectors—understudied but critically important issues for epidemiology. Studying these exposure-related effects is of paramount importance for predicting how they respond to insecticide exposure and whether this exposure makes them more or less likely to transmit pathogens.

**Abstract:**

For many decades, insecticides have been used to control mosquito populations in their larval and adult stages. Although changes in the population genetics, physiology, and behavior of mosquitoes exposed to lethal and sublethal doses of insecticides are expected, the relationships between these changes and their abilities to transmit pathogens remain unclear. Thus, we conducted a comprehensive review on the sublethal effects of insecticides and their contributions to insecticide resistance in mosquitoes, with the main focus on pyrethroids. We discuss the direct and acute effects of sublethal concentrations on individuals and populations, the changes in population genetics caused by the selection for resistance after insecticide exposure, and the major mechanisms underlying such resistance. Sublethal exposures negatively impact the individual’s performance by affecting their physiology and behavior and leaving them at a disadvantage when compared to unexposed organisms. How these sublethal effects could change mosquito population sizes and diversity so that pathogen transmission risks can be affected is less clear. Furthermore, despite the beneficial and acute aspects of lethality, exposure to higher insecticide concentrations clearly impacts the population genetics by selecting resistant individuals, which may bring further and complex interactions for mosquitoes, vertebrate hosts, and pathogens. Finally, we raise several hypotheses concerning how the here revised impacts of insecticides on mosquitoes could interplay with vector-mediated pathogens’ transmission.

## 1. Background

Vector-borne diseases can cause severe harm to human health, including morbidity and mortality depending on the pathogen infection, diagnosis, and treatment quality available for infected individuals [1,2]. For years, the scientific community has worked to develop ways to mitigate the effects of these diseases, and one of the main approaches used is the reduction of vector populations [2,3]. The principal vectors of many human pathogens are mosquitoes. For example, the mosquito *Aedes aegypti* (Diptera: Culicidae) is capable of transmitting multiple pathogens, including the Dengue, Zika, and Chikungunya viruses [4,5]. Furthermore, several mosquito species of the genus *Anopheles* are responsible for transmitting the protozoan *Plasmodium*, which causes malaria, as well as the worms that cause lymphatic filariasis. These worms are also transmitted by other mosquito species, including *Culex quinquefasciatus* (Diptera: Culicidae). The abilities of these pathogens to be transmitted by widely distributed vectors explain their worldwide distributions. For instance, the Dengue virus is now present in at least 129 countries and was estimated to infect around 390 million people every year [1]. Furthermore, the *Plasmodium*-caused disease malaria was considered endemic in at least 87 countries, with 229 million cases reported for 2019 in the latest 2021 report [2].

Several approaches have been used to reduce vector populations, which spans from the reductions of breeding sites up to the use of insecticides to control the abundances of both the vector’s immature and adult stages [6,7,8]. Pyrethroids, synthetic analogs derived from pyrethrins (naturally occurring compounds present in the flower buds of certain *Tanacetum* species), are the most widely used group of insecticides, [9,10]. Pyrethroids and pyrethrins act by disrupting the functioning of voltage-gated sodium channels in insects. Pyrethroids are stabler and more toxic to insects than pyrethrins are and cost less to produce [8,11,12]. Organophosphates and carbamates are two other nerve-active insecticide groups that target acetylcholinesterase enzymes, and consequently are as quick-acting as pyrethroids [13]. Finally, two other relatively slow-acting groups of insecticides commonly used against mosquitoes are insect growth regulators (e.g., pyriproxyfen, which attacks the hormonal balance to disrupt growth and development) and biorational insecticides (e.g., *Bacillus thuringiensis* var. *israelensis* (*Bti*), which targets the midgut) [14,15].

To control adult mosquitoes, several insecticide application techiniques can be used, such as indoor residual sprays (IRS), long-lasting insecticidal nets (LLIN), aerosol sprays, and fumigations [7,8,16]. The IRS and LLIN are the most commonly used methods for the control of *Anopheles* spp., and were responsible for significant decreases in the number of malaria cases in Africa from 2000 to 2019 [2,6,8,17]. However, successive reports of insecticide resistance, especially to pyrethroids, have caused uncertainty regarding the current progress in vector control and put the sustainability of the continuous use of IRS and LLIN in doubt [6,8,16]. Despite the promising launch of a large-scale pilot vaccination program with a first vaccine candidate for malaria in 2019 [17], the report of an increase of 12 million malaria cases per year from 2014–2019, is an indication of delayed progress in malaria control [2]. Pyrethroids are also used to control *Ae. aegypti* and other culicid mosquitoes, and this has unsurprisingly imposed strong pressure for the selection of resistant populations [18,19,20,21,22]. Resistance has also been reported against other insecticide groups, including organophosphates, insect growth regulators, and *Bti* [23,24]. However, since research on the effects of these compounds has been scarce, they have been discussed to a lesser extent than pyrethroids in this review. Interestingly, despite growing reports of pyrethroid resistance globally and the intensive use of insecticides against mosquitoes, the ways in which sublethal exposure to these compounds and resistance-associated population genetic changes affect the transmission of pathogens have remained elusive [25,26].

**Figure 1:**
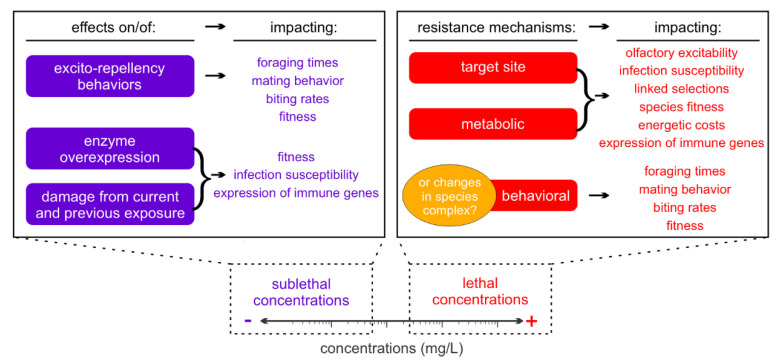
Summary of potential impacts of different insecticide concentrations on mosquito physiology and behavior.

In the current review, the multiple facets of insecticide exposure effects on mosquitoes (Culicidae) that can interplay with their vector competence (i.e., the ability of a vector to transmit a pathogen [27], correlated with overall pathogen transmission risks) were highlighted (Figure 1). The main text is divided into Section 2 and Section 3. Section 2 contains information on the direct and acute effects of sublethal concentrations on individuals and populations; it is further divided into two parts, each dedicated to effects of exposure on adult and larval mosquitoes. Section 3 reviews the effects of changes in population genetics caused by selection for resistance after exposure to high doses of insecticides. Section 3 is organized into three subsections, each discussing one of the three major resistance mechanisms: target site mutations, metabolic resistance, and behavioral resistance. Understanding both the sublethal and lethal effects of insecticide exposure on the biological and behavioral responses of mosquitoes, especially those impacting their blood meal-related activities—a key point in pathogen transmission—can lead to the development of novel approaches that provide comprehensive conclusions linking control strategies to epidemic risks.

## 2. Changes in Pathogen Dissemination by Mosquitoes That Survived an Insecticide Exposure

Insecticides, like any other xenobiotics, can directly impact both the biology and behavior of mosquitoes, after non-lethal exposures. Upon contact with the insects, insecticide molecules can induce behaviors, or cause energy-consuming biochemical reactions before causing lethality [28]. These behaviors or energetic costs can reduce insect fitness by disturbing feeding or reproductive behaviors, and thus interfere with the vector competence [29,30]. For example, only sufficiently “aged” female mosquitoes can transmit the malaria-causing protozoan *Plasmodium falciparum*, which takes about 10 to 15 days to complete its life cycle within the vector and be ready for transmission to humans [26]; therefore, decreases in vector survival time resulting from sublethal insecticide exposure or lower blood meal volumes would reduce pathogen transmission [29,31]. 

Herein, we structured separate sections for different responses of the immature and adult life stages. In the case of adults, two different situations resulting in sublethal exposures have been considered: (a) reduced exposure on treated surfaces because of irritability (i.e., stimulus-dependent repellency) [32,33,34]; and (b) exposure to low insecticide concentrations due to the expected reduction of insecticide residuals in IRS and LLIN [35]. Regarding the sublethal exposure of larvae, sublethal exposures are results from the direct application of insecticides that dilute in water bodies [36,37] or through indirect contamination of aquatic systems by insecticides in runoff [38]. Nevertheless, for both cases (i.e., larvae and adults), most of the impacts of exposures to sublethal concentrations occurred through the toxicity of the insecticide itself and its cascading effects on subsequent generations [39,40]. These sublethal responses contrast with the impacts of lethal exposures, which selected for resistant populations (discussed in Section 3) and thus had presented long-term and stable population genetic effects, even if insecticide use is suspended [32,41].

### 2.1. Sublethal Exposure of Adults to Insecticides

As mentioned above, pyrethroids in IRS and LLIN have been the most commonly used methods for controlling adult mosquitoes. Pyrethroids target voltage-gated sodium channels in insects. They act by prolonging their opening state and causing repetitive firing (type I pyrethroids) or long membrane depolarization (type II pyrethroids); both lead to convulsions, subsequent paralysis, and eventually death [11,12]. Structurally, type II pyrethroids harbor an α-cyano moiety at the phenyl benzyl alcohol position that is absent in type I pyrethroids. The presence of this α-cyano moiety confers type II pyrethroids higher toxicity than type I pyrethroids [9].

In addition to the pyrethroid lethal effects, several mosquito species (e.g., *Anopheles* spp. and *Ae. aegypti*) are irritated by contact with pyrethroid molecules [34,42,43,44]. Studies showed that certain populations of *Anopheles* species also respond to the presence of pyrethroids (especially type I, which are often more volatile than type II) before making physical contact with a treated surface, suggesting their ability to recognize volatile particles by olfaction [33,45]. These excito-repellent behaviors allow sublethally exposed mosquitoes to escape from insecticide residues [43]. This escape behavior could lead to the reduction of human bites inside homes, which would reduce pathogen transmission rates. However, the outcome of this irritability on mosquito physiology and the potential risk of mosquitoes being present in greater numbers outside of homes, where people cannot be protected by bed nets or IRS, should also be considered as potential factors increasing the transmission risks [44].

Mosquitoes’ foraging and learning abilities depend on the normal functioning of their neural system, which can be modulated by both their physiological status and environmental cues [46,47,48,49,50]. Therefore, the direct impacts of sublethal exposures to pyrethroids on most of the insect’s abilities is expected to be dependent on their sensory and neural systems, especially considering that not only pyrethroids but also several other insecticides target the mosquito nervous system [8]. Cohnstaedt and Allan [51] demonstrated that female mosquitoes of *Ae. aegypti*, *Cx. quinquefasciatus*, and *Anopheles albimanus* needed more time to initiate a flight response to host cues, flew slower, and had higher flight turning rates after being exposed to two pyrethroids (i.e., deltamethrin and permethrin, type II and I, respectively). This impaired flight ability is expected to interfere with insect mobility and reproduction, but it is not known to what extent this impairment may affect the transmission of pathogens.

When considering the reproductive and olfactory abilities of insects, the impacts of sublethal concentrations of insecticides were usually reported to be negative for their fitness [52,53,54,55]. However, studies had also reported the absence of these negative impacts, and in less common circumstances, the occurrence of beneficial effects for the insect’s fitness [56,57,58,59,60], the latter which is termed ‘hormetic effect’, and are usually but not exclusively related to pyrethroids [61,62]. Interestingly, the majority of the records of sublethal effects in mosquitoes have demonstrated only negative results after insecticide exposures, with no hormesis identified [35,63]. The fact that there are few studies reporting impacts of pyrethroid sublethal exposure in mosquitoes, and that even in these studies the doses used are high enough to cause mortality of at least part of the exposed individuals (e.g., LD_10_ or higher [63]) might explain the lack of evidence for pyrethroid mediated hormetic effects, which is usually reached at much lower and narrow concentration ranges.

Several species have been found to constitutively overexpress detoxifying enzymes as a tolerance or resistance mechanism [64,65,66,67]. However, the overexpression of such enzymes can also occur only after contact with sublethal concentrations of insecticides in susceptible individuals. This phenomenon of increased expression of detoxifying enzymes was observed in a susceptible population of *Anopheles coluzzii* after sublethal exposure to pyrethroids [68], and this is probably a common response among insects [69,70,71]. This overexpression of enzymes leads to energetic costs that may negatively impact several life-history traits related to fitness. However, the fitness costs of induced metabolic responses in mosquitoes have only been assessed for constitutive metabolic resistance, as mentioned in the next section (i.e., Section 3.2) [72,73]. Additionally, an impaired mosquito would probably have its flight abilities reduced, as it had been shown for other insect species [51,74,75,76], and have most of their biological and physiological parameters impacted.

Pathogens, such as the Dengue and Zika viruses, need to overcome the midgut barrier to disseminate throughout the insect hemolymph and eventually reach the salivary gland before they can be transmitted. Sublethal exposure to a pyrethroid insecticide (i.e., bifenthrin) increased the amount of Zika virus that passed through the midgut of *Aedes albopictus* females [77]. The higher dissemination ability in exposed females may be related to differential energy resource allocation caused by insecticide exposure that reduces the insect imme response, facilitating the dissemination of the virus. Another study showed that this increased dissemination of pathogen happens in the early days after the ingestion of infected blood, but in later days after an infected blood meal, the viral dissemination in the control infected mosquitoes also increases, and both mosquito groups end up presenting 100% viral dissemination [78]. Further studies are needed to confirm the underlying mechanisms that drive the observed differential pathogen dissemination rate found by these studies and to assess how would insecticide exposure impacts pathogen dissemination within mosquito and transmission risks.

Finally, *Bti*, which is normally used in larval control, appears to influence adult *Ae. albopictus* to detect cues in the water body to which *Bti* is added, since its presence induced an increase in the oviposition behavior of this mosquito [79]. However, in the same study, it was demonstrated that the toxicity of *Bti* to emerging larvae could be maintained for several days without any change in efficacy [79].

### 2.2. Sublethal Exposure of Larvae to Insecticides

Many studies that investigated the effects of sublethal concentrations of pesticides on larval mosquitoes have reported that the physiology of an individual is not reset during metamorphosis to enable it to negate these effects in adults [80,81]. The effects of physiological stressors during the immature developmental stage on the adult can also be difficult to interpret, as they may have multiple outcomes, such as longer development times, male-biased sex ratios, and higher emergence rates and body sizes [82,83,84,85] or they offer null impacts, depending on the xenobiotics [86,87]. 

Sublethal exposures to both pyrethroids or organophosphates in the fourth larval instar caused reductions in adult longevity, fecundity, and wing length in *Cx. quinquefasciatus* [82]. Furthermore, in this case, the fewer eggs laid by treated females were also smaller than the eggs of control females [82], which would impact the next generation’s fitness as well. These impacts may be mediated by changes in larval swimming behavior induced by sublethal exposure to these pesticides, as was demonstrated in *Ae. aegypti* [37]. In addition to energy loss due to faster wriggling movements, the impact on the feeding ability of these insects would also explain the longer developmental period necessary to reach the pupal stage [37]. On the other hand, sublethal exposure to malathion in first-instar larvae of *Ae. aegypti* resulted in larger adult females at 20 ºC, but not at 30 ºC, demonstrating that other environmental factors during exposure can also shape the nature of the effects of sublethal insecticide exposures [88]. In this case, the reduced competition resulting from the elimination of small or more susceptible larvae was proposed as an explanation for the larger-sized females that developed from larvae grown at the lower temperature [88]. Nonetheless, the vector competence of the larger adult females originating from larvae grown at the lower temperature was not modified compared to that of the control, while the females originating from exposed larvae grown at the higher temperature presented a significantly higher vector competence for the tested Sindbis virus (MRE16-strain) infection and dissemination rates than the control females [88]. The mechanism underlying the higher adult vector competence of these sublethally exposed larvae could not be assessed in that study, but it was speculated that impairment of the immune system was involved in the increased vector competence in smaller adults [88]. 

Lingering damage to tissues—such as those of the midgut, as a result of larval or adult sublethal exposures to insecticides—is common in insects, including mosquitoes [81,89,90,91]. Because the insect midgut is a port of entry for most pathogens and is thus a barrier to be overcome before the pathogen can infect new hosts [92], any damage to the midgut or the peritrophic matrix (an extracellular matrix that surrounds the food bolus and is synthetized by the posterior midgut after a blood meal) could change a pathogen’s ability to disseminate throughout the insect’s body. *Bti*-based insecticides are normally used for larval control; upon ingestion, these insecticides damage midgut cells. Sublethal exposure to *Bti*-based products increases the susceptibility to dengue virus infections, but not to chikungunya in *Bti*-resistant *Ae. aegypti* [93]. It remains to be elucidated as to whether this is a common effect and related to the sublethal damage by *Bti* on the midgut, or is linked to genetic factors (since it was observed in a *Bti*-resistant mosquito population). On the other hand, the damaged midgut impairs the mosquito’s digestion ability, inducing it to take smaller blood meals [81,89], which conversely reduces the likelihood of mosquitoes initially acquiring the pathogens.

Studies have been carried out considering the interaction between adult mosquitoes derived from exposed larvae and malaria-causing parasites [86,87,94]. For instance, the exposure of *Culex pipiens* to the neonicotinoid imidacloprid did not affect the life history traits of individuals nor the susceptibility of adults to infection by avian malaria parasite (*Plasmodium gallinaceum*) [86]. The exposure of larvae of *Cx. pipiens* to field-realistic doses of glyphosate, the most used herbicide worldwide, did not affect the individual survival, adult size, and female fecundity. Conversely, females derived from exposed larvae with the herbicide increased the probability of female infection by *Plasmodium relictum* [87]. The sublethal exposure to permethrin at larvae or adults of *An. gambiae* reduced the infection prevalence by *Plasmodium berghei* [94]. These studies with laboratory-consolidated models pointed that the level of interference of the exposure of the larvae to insecticides in the process of infection of mosquitoes depends on the species of both vector and pathogen and the mode of action of the compounds. Nevertheless, more realistic field studies are needed to better understand how larval exposure can interfere with the life cycle of pathogens in adult mosquitoes.

## 3. Changes in Pathogen Dissemination in Insecticide-Resistant Mosquitoes

Insecticides often do not reach 100% efficacy at controlling any given target species. Even when applied at recommended field rates, some compounds fail to reach the targeted insects because of biotic and abiotic factors associated with a surface covering failures, compound degradation, or runoff after heavy rainfalls [95,96]. Furthermore, it is not uncommon in any species that there are individuals capable of behaviorally avoiding such compounds or equipped with physiological tools capable of mitigating these compounds’ actions. The result of these factors is that the applied insecticides will almost always leave some survivors. The individuals that survive such insecticide exposures by behavioral means or physiological mechanisms will then reproduce and transfer the traits that permitted their survival to their offspring, increasing the percentage of resistant individuals in the population across generations [32].

The two main classes of insecticide resistance mechanisms are alterations of target sites (e.g., the mutation in the voltage-gated sodium channel gene, for pyrethroids, or in the acetylcholinesterase gene, for organophosphates and carbamates), which generally reduces the binding rate of the insecticide and its target [19,97], and modifications of the insect’s metabolism, which can occur via a variety of detoxification and excretion processes [98]. Both of these insecticide resistance mechanisms (i.e., target site modification and changes in metabolism) occur in mosquitoes. Furthermore, as already described for other insect groups [99,100,101,102,103], attention has recently been paid to behavioral resistance mechanisms to insecticides in mosquitoes [6,104,105]. The selection of resistance through any of these mechanisms will shape the genetics of mosquito populations and could impact insect physiology and behavior, and therefore transmission abilities.

### 3.1. Target Site Insecticide Resistance

Most known cases of insecticide resistance related to target site alterations in mosquitoes involves knockdown resistance (*kdr*) mutations in the insect sodium channels, which are the major targets for the actions of pyrethroids [8,97,105,106]. The occurrence of *kdr* mutation, which reduces the action of insecticide molecules targeting voltage-gated sodium channels, was shown to also cause changes in the gating properties of such ionic channels [97,107,108,109]. Therefore, it is reasonable to expect modified firing activities (i.e., low action potential frequency) in some neural circuits of individuals carrying *kdr* mutations, potentially resulting in differential sensitivity to environmental cues.

Considering that a large part of a mosquito’s host-seeking behaviors depends on olfactory sensory neurons [48,49], and that blood meal intake behavior has also been suggested to be modulated by specific sensory neurons in the tip of the mosquito stylet [110], modified neural activities in *kdr*-mutant mosquitoes could have important impacts on insect fitness. Diop et al. [111], for example, demonstrated that L1014F *kdr* homozygous *An. gambiae* had an impaired ability to locate holes in bed nets, suggesting a decrease in their overall sensory abilities due to this mutation. Another study also showed that an L1014F *kdr* insecticide-resistant strain of *An. gambiae* preferred hosts under insecticide-treated nets more than those under untreated nets, while isoline-susceptible mosquitoes did not discern between both netting options [112]. This preference for insecticide-protected hosts suggests that the L1014F *kdr* mutation in the voltage-gated sodium channels modulates the mosquitoes’ host preference towards insecticide presence.

Differential susceptibility to pathogen infection has been reported in mosquitoes harboring *kdr* mutations, independently of insecticide exposure [113,114,115]. These studies indicated greater susceptibility of L1014F and L1014S *kdr* mutants of *An. gambiae* to infection by *P. falciparum*, which in turn represents a worsening scenario for malaria control in regions where pyrethroid insecticides are used heavily and mosquitoes have already evolved pyrethroid resistance [113,114,115]. Nonetheless, another study showed that even though L1014S *kdr* increased the susceptibility of mosquitoes to *P. falciparum* when pyrethroids were present, the insecticide had toxic effects directly on the pathogen, thus reducing the overall infection risk [116]. This suggests that the higher mosquito susceptibility to parasite infection is compensated for by the toxicity of the insecticide to the parasite itself.

When a mosquito harboring a given *kdr* mutation is selected, several other polymorphisms located in the vicinity of the voltage-gated sodium channel locus might also be selected for, even under weak selective pressure, and thus exponentially increase in the population [117]. These new frequencies of a certain haplotype might cause slight to strong changes in the mosquito’s physiology (including its vector competence) or behavior. Previous investigations reported a positive correlation between high frequencies of a specific haplotype for the immune gene *ClipC9* and high frequencies of the L1014F *kdr* mutation, indicating that linked selection could be playing a role in shaping genetic traits other than the one under direct selective pressure [115]. When this immune gene was inhibited in the L1014F *kdr*-mutant *An. gambiae*, their susceptibility to *P. falciparum* increased, demonstrating the immune gene’s direct role in controlling the mosquito’s susceptibility to this pathogen. This immune gene was also shown to be located in a locus very close to the voltage-gated sodium channel gene [115]. This would, therefore, explain the multiple different outcomes of *kdr* mutations observed in several different populations, as these might be linked to different haplotypes of other genes that have indirect positive or negative impacts on the transmission rates, thus making the nature of these impacts hard to predict.

Another set of studies have also investigated a target-site mutation in the acetylcholinesterase gene (i.e., *Ace-1* G119S) that confers resistance to organophosphates and carbamates [22,118]. Given the involvement of this enzyme in the neural signaling, the same broad scenario of impacts of altered activity as results of mutations that were discussed for *kdr* resistance could be expected. However, at least one study provided evidence that the biochemical activity levels of this enzyme do not change with the mutation [119]. This is the only mutation found in this gene in both *Anopheles* and *Culex* and the selection process seemingly selected a single haplotype (*Ace-1^R^*) with signals of linkage selections across at least two megabases in the genome of *An. gambiae* [120]. The reduced diversity across the genome of the resistant individuals therefore suggests a source for the several fitness costs and adult behavioral disadvantages reported by literature [120,121]. Although the specific pathways in which the selection for *Ace-1^R^* allele can impact vector competence is not well known yet, its negative impact on fitness does not necessarily imply a lower transmission risk since higher *P. falciparum* infection prevalence was also described to occur in the resistant individuals compared with susceptible ones of same genetic background [122].

Insecticide resistance could also indirectly interfere with overall vectorial capacity, which is influenced by variables such as vector density and longevity as well as transmission of pathogens ([123] and references therein), by causing changes in insect fecundity, mainly through modulations to reproductive abilities by modifying mating or blood meal feeding abilities in resistant individuals. The previously mentioned changes in neural excitability may also directly impact the female-male communications and mating success of *kdr or Ace-1^R^*-expressing individuals, or even impact the blood volumes that these females take to produce mature, viable eggs. Platt et al. [124] showed that a L1014F *kdr* mutation in *Anopheles* spp. benefitted mating competitiveness when in heterozygosis, but in homozygosis this mutation reduced mating competitiveness. Another study also showed the mating disadvantage for *An. gambiae* males carrying the *Ace-1^R^* allele [121]. This observed effect on mating success could be caused by changes in neural olfactory perception of sexual aggregation pheromones, as previously demonstrated in *Ae. aegypti* [125], but further investigations are needed to confirm this hypothesis.

### 3.2. Metabolic Insecticide Resistance

Metabolic resistance in mosquito species usually involves increased expression of enzymes (e.g., esterase or monooxygenases dependent on cytochrome P450) associated with detoxification processes [64,65,66,67,126]. Unlike target-site mutations, the genetic mechanisms underlying metabolic resistance are more easily and logically linked to direct energy losses, since in most cases resistant insects possess higher expression levels of resistance enzymes, which surely consumes energy [73,127]. These energetic costs could indirectly or directly affect components of insect fitness (e.g., fecundity and longevity), pathogen transmission rates, and overall population densities [128,129]. Additionally, the regulatory mechanisms involved in the overexpression of detoxification genes could also select for other linked gene variants, as was discussed in the previous section [115].

The studies on the fitness costs of metabolic resistance in insect pests showed multiple different outcomes, which could be explained by differences in experimental designs or laboratory versus field conditions [41]. Nevertheless, the overexpression of both esterase and cytochrome P450 conferring resistance to two different insecticide groups (i.e., organophosphates, and pyrethroids) in *Cx. pipiens* reduced its energy reserves (e.g., glycogen, glucose, and lipids) by up to 30% [72,73]. In P450-overexpressing insects, smaller-bodied females and lower female emergence rates [72] were observed, which is suggestive of lower fecundity and potential population decreases, similarly to those demonstrated in organophosphate-resistant beetles overexpressing esterase enzymes [130]. These lower energy resources could also cause shorter adult lifespans, which could reduce the pathogen transmission risks, as was demonstrated for *Cx. pipiens* overexpressing esterase enzymes [131].

The effects of the overexpression of detoxification enzymes on the transmission rates of pathogens are still controversial. It has been suggested that esterase overproduction can interfere with *P. relictum* development in *Cx. pipiens* [132]. However, more recent studies have suggested that this resistance mechanism decreased the susceptibility of *Cx. pipiens* to infection by this pathogen [133]. This reduction in infection rates more likely resulted from the increased expression of multiple immunity-related genes than from changes in energy costs [133]. This linked overexpression of multiple immune-related and detoxifying genes indicates that the same regulatory mechanisms and gene expression profiles are shared between these gene groups [117,133], suggesting that metabolic resistance can indirectly cause other physiological changes, including changes in vector competence.

The mechanisms involved in regulating the expression of detoxifying enzymes by the downregulation of microRNAs (i.e., short RNA sequences that bind to specific regions of their target mRNA to prevent protein synthesis) have recently been elucidated [134]. The downregulation of four of these post-transcriptional specific inhibitors of enzyme synthesis leads to higher levels of the cytochrome P450 enzymes that confer resistance to pyrethroids in *Cx. pipiens* [135,136]. These microRNAs are present in the genome in clusters, and their downregulation could impact several other physiological systems in the mosquitoes and interfere with their pathogen transmission abilities.

The overexpression of detoxifying enzymes might also interfere with mosquito host-seeking behaviors by disrupting their olfactory abilities. It is well-known in insects that for correct flight navigation towards an odorant source, every odorant molecule must be degraded after targeting its odorant receptor to allow the signal to be interrupted and new molecules recognized [137]. These processes are largely performed by enzymes, which are mainly esterase and cytochrome P450 enzymes [137,138]. Interestingly, members of a large class of cytochrome P450 genes (e.g., CYP3 and CYP4) that are strongly related to insecticide and other plant xenobiotic detoxification processes were also shown to be expressed at high levels in the antennae of herbivorous pests [138], suggesting a link between these two physiological functions (i.e., metabolism of insecticides and odorant compounds). Therefore, it is not known if high expression levels of esterase or cytochrome P450 enzymes could also metabolize odorant molecules faster in insecticide-resistant mosquitoes. The potential interference with host-seeking behavior in metabolically resistant mosquitoes would reduce their blood feeding frequency, fitness, and thus, pathogen transmission rates.

### 3.3. Behavioral Insecticide Resistance

The behavioral resistance discussed in this section relates to the failure of the insecticide to control the mosquito population because the mosquitoes are repelled by the insecticide and avoid exposure to it. This concept of behavioral resistance in mosquitoes has become a topic of concern due to the long-term, intensive use of IRS and LLIN over the last decades. Mosquitoes have recently been reported to show stronger repellent behaviors to pyrethroids than previously observed [6,33,139].

Since repellent behaviors to an insecticide directly change the exposure rate/time, their potential impacts on vectorial capacity through interactions with insecticides are very complex. Repellent behaviors can easily modulate the amount of insecticide to which an insect is exposed, therefore changing the dose it experiences from lethal to sublethal, and can also modulate the selection dynamics of physiological resistance to insecticides in different ways. We propose the following three main scenarios, considering the target site for the repellency is different than the one for lethality: (1) If a genotypic variant conferring insecticide repellence is already present in a population at a high frequency, this will decrease the likelihood of a physiological resistance mechanism being selected, since many susceptible individuals will survive exposure by being repelled. (2) If physiological resistance was selected before a repellent genotype arose in the population, then repellence is less likely to be selected since many resistant but non-repelled insects also survive. (3) Finally, when both variants are present in the population but not yet at high frequencies, they would reduce the selection pressure for each other, slowing down the selection for both genotypes, keeping their frequencies more stable until other factors disrupt such an equilibrium [32,140,141].

Transfluthrin, a pyrethroid, can cause behavioral repellency in susceptible *Ae. aegypti* adults by acting solely on the voltage-gated sodium channels. Transfluthrin-mediated repellency is a likely result of insecticide actions in selected neuronal circuits [142]. Similar repellency mediated by the activation of sodium channels has also been demonstrated for natural pyrethrins [143]. The presence of the two *kdr* mutations (S989P and V1016G) in the sodium channel gene reduced pyrethroid-mediated repellency without impairing olfaction [143,144]. Other studies that used unrelated field-resistant populations have observed indirect effects of apparent linked or fitness-related alterations in repellency behavior to pyrethroids or other repellents in both *Ae. aegypti* and *An. gambiae* [144,145]. Nonetheless, further research is required on whether the observed increase in *Anopheles* repellency behavior to pyrethroids in the field is a direct or linked effect of *kdr* or metabolic pyrethroid resistance (with respect to lethality).

**Figure 2:**
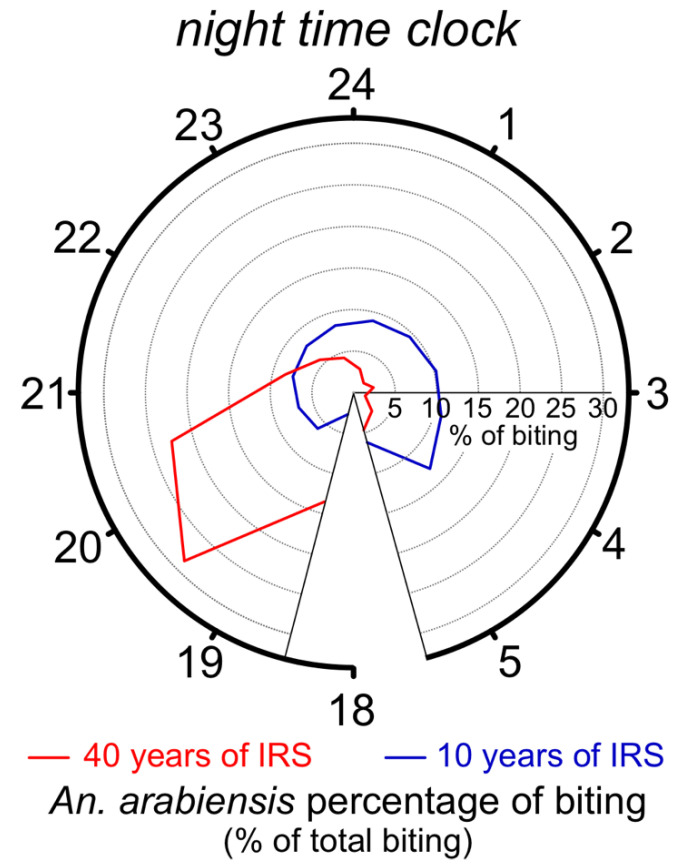
Time-related foraging behavioral changes potentially reducing the efficacy of *Anopheles* control. The colored lines within the clock represent the distribution of *An. arabiensis* biting times in places with short- (**blue**) or long-term (**red**) use of indoor residual sprays (IRS). The biting number decreases toward the center of the clock and increases toward the distal region of the clock. The clock indicates only the late afternoon and nighttime evaluation period from 6:30 p.m. (18:30) to 5:30 a.m. (5:30), as no data for other periods of the day were available. Adapted from Dukeen [158] and Yohannes [149].

In addition to direct repellence behavior, the foraging period patterns of mosquitoes after the introduction of IRS and LLIN have also received special attention. Higher rates of mosquito bites in humans reported after the use of residual sprays and treated nets occurred through a temporal shift in mosquito foraging from late night to early evening (Figure 2) when people are still out of their beds or homes [139,146,147,148,149]. This shift could be a result of selections acting on trait variants and/or changes in ecological interaction among closely related species, especially in the case of the *An. gambiae sensu lato* species complex.

The existence of selective trait variants in a species leading to differences in endo/exophagy preferences was suggested in a study that detected changes in this foraging preference before and after the use of IRS and LLIN within a single species (i.e., *An. gambiae sensu stricto*) [150]. The behavioral trait that these selected phenotypes variants harbor (endophagy or exophagy) could be a result of single or multiple mutations in specific genes related to the circadian rhythms that influence time-specific behaviors, as has been observed in butterflies and drosophilids [151,152]. Additionally, changes in ecological interactions as a result of decreases in the density of one sibling species in the community that was lethally exposed to an insecticide, which benefitted and increased the prevalence and success of other species, were suggested by several studies. For instance, changes in species composition of *An. gambiae s.l.* occurred with a reduction in the density of more endophilic species (e.g., *An. gambiae s.s.*) and an increase in the density of more exophilic ones (e.g., *Anopheles arabiensis*) [147,153,154,155]. Similar shifts toward exophilic behavior at both the inter- and intraspecies levels have also been reported for the *Anopheles funestus* s.l. complex [147,156,157].

The above-mentioned ecological selectivity of insecticides for species with higher relative numbers of exophilic mosquitoes would benefit the adults of the exophilic species by reducing the overall larval density, and therefore, the competition stress experienced by its larvae. In turn, this could also cause an increase in the absolute number, size, and fitness of the adults of the remaining exophilic species [159]. The larger size of adults is related to there being longer-lived females in the population, which increases the pathogen transmission risks [31,160]. Therefore, the ecological selectivity of an insecticide, in addition to shaping the species assemblage towards individuals that are more exophilic or exophagic, may also induce higher mosquito fitness and pathogen transmission risks.

**Figure 3:**
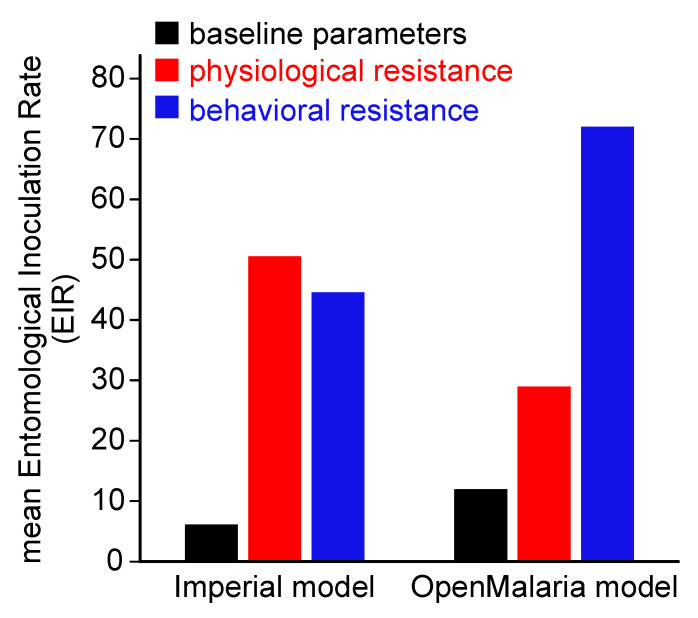
Malaria transmission model predictions as impacted by insecticide resistance after the use of LLIN and IRS. LLIN: long-lasting insecticidal nets. IRS: indoors residual spray. EIR: entomological inoculation rate (infectious bites per person). Adapted from Gatton et al. [6].

A higher prevalence of host-seeking in the early morning hours represents a greater risk of exposure of humans to biting mosquitoes [6,139,150]. Independently of the specific mechanism involved (e.g., inherited behavioral resistance, species assemblage changes, etc.), these behavioral changes could increase the pathogen transmission rates to similar or higher levels than target-site or metabolic insecticide resistance would, as has been demonstrated by two different mathematical models (i.e., Imperial and OpenMalaria models [161,162]) of malaria transmission. These two models predicted the effects of the increasing frequency of exophagy after the use of IRS and LLIN on mean entomological inoculation rates (infectious bites per person) (Figure 3) [6]. Therefore, more studies are needed to understand the very complex scenario resulting from the multiple species and multiple effects of the lethality of insecticides in these vectors in the real world.

## 4. Conclusions

A broader understanding of the effects of insecticides on vectors of human pathogens is needed to support continuous efforts aimed at epidemic reductions. Every year, new disease outbreaks occur, such as the Zika epidemic outbreak in 2015 in South America and the increasing number of cases of Dengue infection worldwide. These outbreaks point out the fact that, despite causing high mosquito mortality, the sublethal effects mediated by insecticides on the behaviors and physiology of mosquitoes can also influence their transmission of pathogens. A large number of studies have suggested that insecticide resistance has modified the physiology, blood-feeding behavior, and reproduction of mosquitoes, and to some extent the dynamics of many diseases that vector mosquitoes can spread. The present comprehensive review and discussion on how unintentional selection for insecticide resistance can drive the overall transmission risks of pathogens by different heritable traits and mechanisms in mosquitoes could help us to better predict, understand, and mitigate common and unexpected epidemics like those that have occurred recently.

The effects of sublethal exposures, on the other hand, involve even more dynamic environmental and ecological interactions that are much less tractable and reproducible by studies when compared with the effects of insecticide resistance in terms of population genetic changes. Thus, even though numerous studies have been done on these sublethal effects, establishing comprehensive and predictable links between the effects of sublethal exposures and changes in vector competence is still a challenge. Therefore, the sublethal effects of insecticides on mosquito vector competence might still be considered a large research gap, with there being a long way to go before we can obtain a more comprehensive understanding of their effects and mechanisms.
